# Disease Prediction in Cattle: A Mixed-Methods Review of Predictive Modeling Studies

**DOI:** 10.3390/ani15172481

**Published:** 2025-08-23

**Authors:** Lilli Heinen, Robert L. Larson, Brad J. White

**Affiliations:** 1 Department of Diagnostic Medicine and Pathobiology, College of Veterinary Medicine, Kansas State University, Manhattan, KS 66502, USA; lheinen@vet.k-state.edu; 2 Department of Clinical Sciences, College of Veterinary Medicine, Kansas State University, Manhattan, KS 66502, USA; rlarson@vet.k-state.edu

**Keywords:** machine learning, artificial intelligence, health, bovine

## Abstract

Predictive models use historical data to predict a future event and can be applied to a wide variety of tasks. A broader evaluation of the cattle literature is required to better understand predictive model performance across various health challenges and to understand data types utilized to train models. This narrative review aims to describe predictive model performance in greater detail across various disease outcomes, input data types, and algorithms with a specific focus on accuracy, sensitivity, specificity, and positive and negative predictive values. A secondary goal is to address important areas for consideration for future work in the beef cattle sector. In total, 19 articles were included. Broad categories of disease were covered, including respiratory disease, bovine tuberculosis, and others. Various input data types were reported, including demographic data, images, and laboratory test results, among others. Several algorithms were utilized, including neural networks, linear models, and others. Accuracy, sensitivity, and specificity values ranged widely across disease outcome and algorithm categories. Negative predictive values were greater than positive predictive values for most disease outcomes. This review highlights the importance of utilizing several performance metrics and concludes that future work should address prevalence of outcomes and class-imbalanced data.

## Simple Summary

Predictive models use historical data to make future predictions. These tools have become more common in the cattle industry in recent years. Their applications are broad but they are especially useful in the prediction of disease. This review explores published studies that use predictive models to predict health outcomes in cattle. Various data types and health outcomes were investigated to characterize predictive model accuracy. Models performed with low and high accuracies depending on outcome, algorithm, and data types. Several challenges were highlighted including data quality and access and rare disease outcomes. This review shows the importance of using more than one performance metric, using easy-to-collect and informative data, and demonstrates that future work should focus on improving how models handle rare outcomes.

## 1. Introduction

As in any sector of the livestock industry, maintenance of animal health and well-being is a priority in the beef industry. The costliest disease affecting the beef cattle industry continues to be bovine respiratory disease [1]. In addition, other conditions such as liver abscesses affect production and profit despite having no outward clinical signs [2]. Several foreign animal diseases, such as foot-and-mouth disease, threaten the North American industry as well [3]. National databases, on-farm record-keeping servers, and laboratory or clinical testing provide vast amounts of data that could be used for earlier detection of disease or outbreaks. Modern technology makes it easy to access, store, and utilize this data to its full capabilities.

Machine learning and predictive modeling offer powerful tools for the beef industry to aid in the detection of disease and determination of prognosis. Machine learning can incorporate large, complex datasets into algorithms used to predict outcomes and make estimations. By handling large amounts of data with ease, machine learning algorithms can utilize technology to complete tasks that would otherwise require human labor that could be best used elsewhere. Potential increased efficiency makes machine learning and predictive modeling attractive for the beef production sector as labor is a precious resource.

Several reviews have described the literature on predictive modeling in various cattle production systems [4,5,6,7,8,9]. Reviews from the dairy sector summarize the capabilities of machine learning to assist in measuring performance outcomes as well as disease detection and health monitoring [4,5,7,8]. Predictive modeling in the beef sector focuses largely on bovine respiratory disease [6,9]. Other diseases or conditions affecting performance are under-represented in these literature reviews. The objective of this review is to report the performance of supervised machine learning algorithms predicting health outcomes for respiratory disease, bovine tuberculosis, foreign animal disease, and others in cattle production systems while describing input data, algorithms, and the hierarchy of the outcome variables to determine areas of consideration when utilizing predictive modeling in the cattle production sector.

## 2. Materials and Methods

For this review, a mixed-methods approach was utilized. A systematic approach was used to search the literature and include relevant articles. A narrative approach was used to summarize the results of selected articles and identify areas of consideration for usage of predictive models in cattle production. The PubMed [10], CAB Direct [11], and Agricola [12] search engines were used. These databases were selected to ensure adequate coverage of primary research in the beef and dairy sectors of the cattle industry.

Initial search terms were selected based on the objective statement: cattle, dairy, beef, machine learning, and disease. Search terms were entered into the MeSH database from PubMed [10] to add synonyms and preferred search words. This resulted in the following search string: (((cattle) OR (dairy)) OR (beef)) OR (feedlot)) OR (bovine)) AND ((((predictive learning model) OR (machine learning)) OR (artificial intelligence)) OR (deep learning))) AND (((disease) OR (morbidity)) OR (mortality)). This search string was used to search the databases in the article title, abstract, and keyword fields. Years of publication were not restricted.

Inclusion and exclusion criteria were applied to select articles relevant to the systematic literature review objective statement. The criteria are displayed in Table 1. Two authors (LH and BJW) evaluated the article titles and abstracts based on these criteria. If a consensus could not be reached, the article was evaluated by a third author (RLL) to break the tie.

Following the review process, each selected article was read in full to extract relevant data. The data were recorded in an Excel spreadsheet and structured, so each row represented a “study arm” from a selected article. A study arm serves as the smallest unit within an article for which performance metrics are reported. Study arms could represent different algorithms, different outcomes, or different data inputs. The columns represented data points extracted to evaluate the systematic review objective. Table 2 describes study arms in greater detail. Additional articles were removed at this step; all articles referencing only dairy cattle or calves were removed from consideration. While healthy calves represent a fundamental attribute of any cattle operation, newborn calves are managed very differently and are physiologically distinguishable from mature bovines, so articles describing machine learning use in newborn calves were not retained. If an article reported using data from both dairy and beef cattle, it was retained. Articles where a clear production system could not be identified were also removed. Furthermore, only articles utilizing machine learning classification algorithms were retained in order to compare predictive model performance across the same metrics; articles describing the use of regression algorithms were removed. Finally, model performance metrics of interest were accuracy, sensitivity, specificity, positive predictive value, and negative predictive value. Articles were removed if these values were not reported or could not be calculated based on data provided in the article.

### Predictive Model Performance Metrics

All of the following measurements were calculated using counts of possible model outcomes: true positive, true negative, false positive, and false negative. A true positive is a unit (animal, group, or operation) that is identified as belonging to the positive class by the model and is truly positive. A true negative is a unit that is identified as belonging to the negative class by the model and is truly negative. A model may also make mistakes and misclassify a unit. A false positive is a unit that the model determines to belong to the positive class but is truly negative. A false negative is a unit that the model determines to belong to the negative class but is truly positive. Using the counts of these model outcomes, one can calculate various metrics for the evaluation of predictive model performance.

Accuracy measures how well the model determines truly positive and truly negative units in the total tested population. Accuracy is calculated by dividing the number of true positives and true negatives by the total population tested using the model. Sensitivity measures the ability of the model to find the truly positive units and is calculated by dividing the number of true positives by the total positive population (true positives and false negatives). Specificity measures the ability of the model to find the truly negative units. Specificity is calculated by dividing the number of true negatives by the total negative population (true negatives and false positives).

Positive predictive value (PPV) and negative predictive value (NPV) are additional measures of model performance and are affected by the prevalence of the outcome of interest. PPV refers to the probability that a positive classification by the model correctly identifies a unit as positive, and it is calculated by dividing the number of true positives by the model’s total positive population (true positives and false positives). NPV refers to the probability that a negative classification by the model correctly identifies a unit as negative. NPV is calculated by dividing the number of true negatives by the model’s total negative population (true negatives and false negatives). Using all these metrics to evaluate predictive model performance is important as the prevalence may affect some metrics more than others. Additionally, an imbalanced dataset can result in the model favoring the prediction of one outcome over the other, resulting in models that can appear to have great accuracy but at the expense of other metrics such as sensitivity or specificity [13].

## 3. Results

The initial search led to a total of 1061 articles across the three search databases (PubMed, CAB Direct, and Agricola). Following the removal of duplicates, 776 unique article titles and abstracts were available for review. Several articles (*n* = 122) were removed from review due to inability to access the full article text or due to the full article text not being written in English. This resulted in 654 unique articles to be evaluated for final inclusion using the inclusion and exclusion criteria displayed in Table 1.

Following the review of 654 abstracts, two authors (LH and BJW) agreed on the inclusion of 118 articles in the final evaluation. The disagreements (*n* = 53) were reviewed by a third author (RLL) and resulted in the inclusion of 27 additional articles. This left a total of 145 articles for full review and data extraction. Removal of all articles referencing dairy cattle or those referencing an unknown production system resulted in removal of 117 and 4 articles, respectively. One article was removed due to describing the use of regression algorithms for prediction. Finally, four articles were removed due to inability to calculate all metrics of interest. The remaining 19 articles were used for data collection.

Of these 19 articles, 11 studies described data collection and model building as occurring only in beef animals. The remaining studies (*n* = 8) were conducted in both dairy and beef systems. Articles were published between 2014 and 2025 in 12 different journals. Several production sectors were included in the 19 articles and consisted of feedlot or other confined feeding operations, cow–calf operations of various sizes, and growing cattle on pasture. Health outcomes of interest covered 13 unique diseases: acute interstitial pneumonia, broncho-interstitial pneumonia, bovine respiratory disease, other types of pneumonia, foot-and-mouth disease, lumpy skin disease, bovine tuberculosis, heat stress, infectious bovine keratoconjunctivitis, liver abscess, anaplasmosis, babesiosis, and total morbidity. These were broadly categorized into four different disease processes (respiratory disease, foreign animal disease, bovine tuberculosis, and other). The hierarchy of the outcomes also varied between articles. An operational-level outcome was defined as an outcome that could be determined at the level of a farm or feedlot. A group-level outcome was considered an outcome determined at the level of a cohort of animals that had all been managed in a similar manner but were part of a larger operation, for example, a pen of cattle at a feedlot. An individual-level outcome was one in which the outcome was determined at the level of a single animal, regardless of that animal’s participation in a group or farm. The majority of articles (*n* = 11) investigated individual-level outcomes. Six studies investigated operation-level outcomes. Only two studies investigated group-level outcomes.

The input data used to train the predictive models were diverse between the investigated articles. To simplify analysis, input data were broadly categorized as follows: images and videos, climate data, laboratory test results, and demographic data. Several studies included input data from multiple categories. Demographic data could further be categorized into individual-level, group-level, and operation-level data. Many studies utilized individual-level input data to predict an individual-level outcome (*n* = 11). Four of those studies also utilized operation-level and group-level data. Operation-level input data alone was used to predict operational-level outcomes in six articles. Only two studies used group-level data alone to predict group-level outcomes. Table 3 describes the input data used by the 19 articles by disease outcome category in greater detail.

The prevalence of health outcomes varied between the studies. Table 4 reports the average prevalence of each health outcome as well as the number of articles and study arms contributing to the prevalence. The range of prevalence for each health outcome is also displayed for the health outcomes for which more than one prevalence was reported. The articles included in the current literature review reported prevalence in addition to performance metrics. This is important as prevalence can impact various performance outcomes. Specifically, positive and negative predictive values (PPV and NPV) will vary depending on a low or high prevalence outcome.

**Figure 1:**
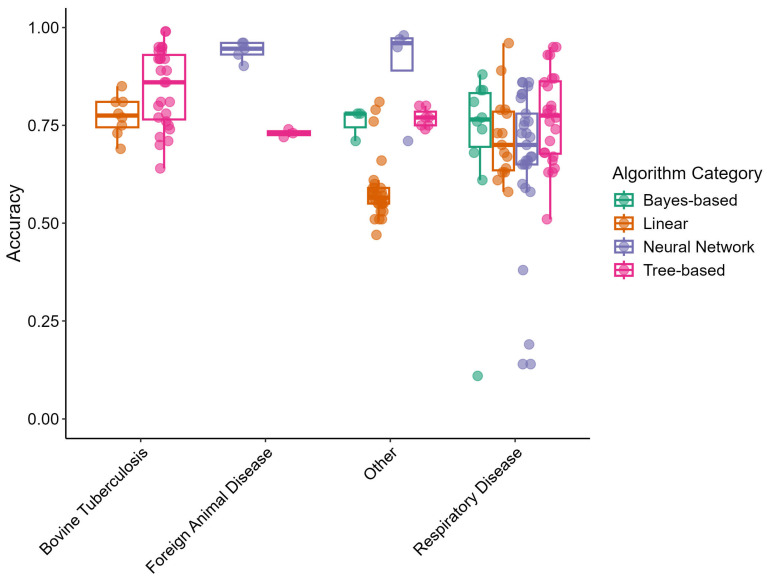
Box-and-whisker plots of predictive model accuracy by disease outcome and algorithm categories extracted from reviewed articles. Points represent independent study arms.

Predictive model performance metrics were evaluated by disease outcome category and by algorithm category. The “Other” and “Respiratory Disease” outcome categories included all algorithm categories. The “Bovine Tuberculosis” disease outcome category only included linear and tree-based models. The “Foreign Animal Disease” category included few articles and only covered neural network- and tree-based models. Figure 1 displays box-and-whisker plots of accuracy for the different disease outcome categories and algorithm categories. Points are used to indicate individual study arms. Accuracies were clustered together among the different algorithm categories in the “Respiratory Disease” outcome but varied in the “Other” category. Non-linear algorithms (neural networks, tree-based, and bayes-based) appeared to perform with higher accuracy than linear algorithms.

**Figure 2:**
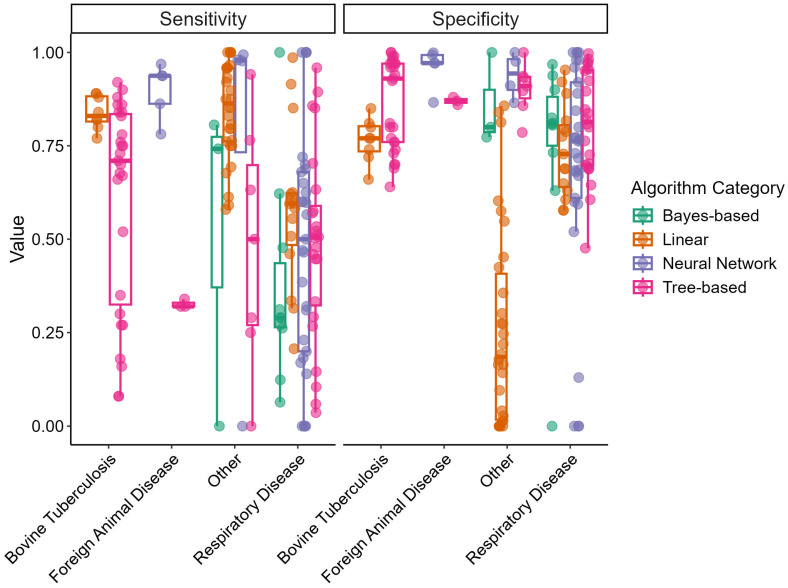
Box-and-whisker plots of predictive model sensitivity and specificity by disease outcome and algorithm categories extracted from reviewed articles. Points represent independent study arms.

Figure 2 displays box-and-whisker plots of sensitivity and specificity for the different disease outcome categories and algorithm categories. Sensitivity varied greatly for all disease outcomes, particularly within the “Respiratory Disease” category. The linear algorithm category displayed the smallest variability in sensitivity among the algorithm categories. Specificity varied less and tended to be in the upper range (>0.50) for all disease categories; however, linear models in the “Other” disease category displayed a wide range in specificity.

**Figure 3:**
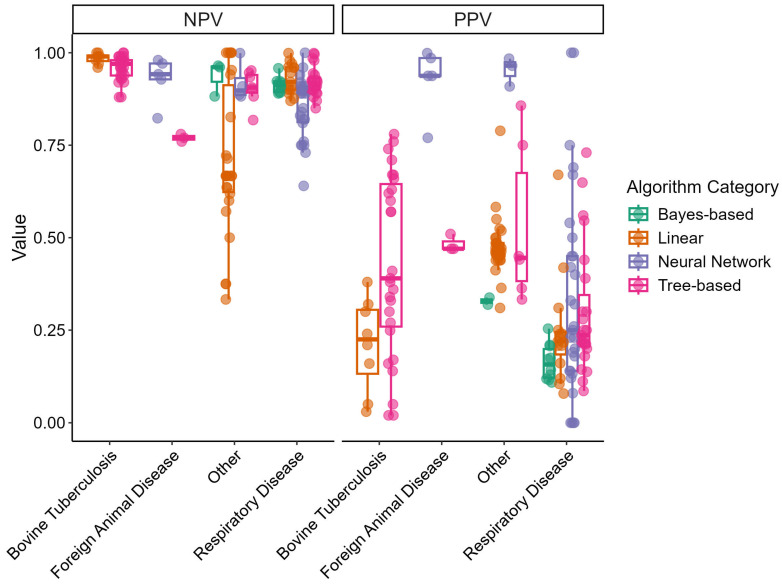
Box-and-whisker plots of predictive model’s negative predictive value (NPV) and positive predictive value (PPV) by disease outcome and algorithm category extracted from reviewed articles. Points represent independent study arms.

Figure 3 displays box-and-whisker plots of the NPV and PPV for the different disease outcome categories and algorithm categories. NPVs tended to be high across different disease outcome categories and displayed little variation. Linear models built for the “Other” disease outcome category demonstrated a wide range of NPVs. Consequently, PPVs demonstrated lower values overall and displayed greater variability. Neural network models in the “Foreign Animal Disease” and “Other” categories displayed high PPVs.

## 4. Application in Beef Cattle

The search terms used in this literature review revealed hundreds of relevant articles. The application of machine learning to production agriculture has become popular in recent years, resulting in the publication of many articles relevant to livestock health conditions. The majority of the 145 articles originally selected for inclusion by the three authors described the application of predictive models to dairy cattle diseases. Data analysis within the dairy industry, especially in North America, benefits from vertical integration, meaning detailed data is often available for individual animals. Additionally, a variety of automatic sensors and computer systems have been adopted by the dairy industry in recent years. The vast amount of data generated by this technology lends itself well to machine learning techniques to detect health incidents or decreased performance of individual cows and contributes to the large amount of research on the application of machine learning in the dairy cattle sector. In the current review, however, on-animal or on-farm sensors are not evaluated as they are categorized as “unsupervised” machine learning aimed more at pattern recognition. The studies included in the current review utilize “supervised” machine learning in which the algorithm is explicitly informed by the user as to which classes should be identified.

There could be several reasons for why machine learning is not as heavily investigated in the beef sector when compared to the dairy sector of the cattle production industry. The lifespan of the animals in question varies between the two. Dairy cows remain in production for several years whereas beef animals are reared in confined feeding operations for several months before slaughter. Therefore, while dairy cows are productive for many years, beef cattle are only productive once. Additionally, dairy cows are more easily monitored on an individual animal basis due to the way the product of the dairy industry is harvested. Conversely, beef animals are typically managed and marketed in groups. An additional challenge in the beef sector is the ability to collect data on animals on pasture. Regular data collection may not be possible in grazing animals and limited internet capabilities may restrict the use of automatic sensors.

Comparatively few studies describe the application of machine learning to health issues in beef cattle. Many articles included in this review focus their efforts on bovine respiratory disease. As this is the costliest and most prevalent condition affecting the beef industry, specifically in the feedlot sector, this makes sense [1]. Bovine tuberculosis is also well-represented in this review. This condition is also widespread and costly, particularly in countries where eradication efforts are underway [14,15]. An additional reason why these two conditions are well-represented in this review could be due to the availability of the data used as inputs for the models predicting BRD and bovine tuberculosis outcomes. For example, the bovine tuberculosis articles utilized national databases on previous testing information collected by government agencies tasked with the tracking of tuberculosis outbreaks in cattle. Many articles investigating the use of predictive models in BRD outcomes utilized treatment data from feedlots.

## 5. Model Input Data

A variety of input data were utilized across the 19 articles included in this review. The most popular category was demographic data [16,17,18,19,20,21,22,23,24,25,26,27,28]. This broad category featured three distinct hierarchies of data: individual animal-level, group-level, and operation-level data. In the beef sector, animals are almost never marketed or managed as individuals except for the treatment of sick individuals. Group-level demographic data describes the characteristics of the group, such as sex, average weight, and origin, among others. These characteristics not only describe the group but also inform various operation-specific management and marketing practices. For instance, weight and sex often determine a group of cattle’s risk level for high morbidity at a feedyard. Operation-level demographic data can describe unique aspects of a single operation but may not be relevant if the predictive model is built for one operation only. The complicated structure of livestock production data lends itself to machine learning, especially non-linear algorithms, which may be better able to model complex relationships between variables. However, special attention must be paid to ensure that the levels of data are used in a manner that does not result in overgeneralization of a model. This becomes an issue when models are trained on the data of one or multiple operations but applied to a different group of operations in which that data is not relevant.

Climate data was utilized by several studies [19,23,24,26,27,28]. Environmental factors likely contribute to the spread of disease and are significant in highly transmissible diseases such as foot-and-mouth disease [29]. Additionally, the association of weather data with BRD morbidity and mortality has been investigated using traditional inferential statistics [30,31]. One article in the current review directly evaluates the effect of the inclusion of weather data on the performance of predictive models [19]. This article concluded that there was no benefit from the addition of weather data. Several studies included climate data but did not compare the model performance of models built with and without it. These studies investigated bovine tuberculosis outbreaks in England. In the context of this outcome, climate data was reported to have high feature importance, indicating that the inclusion of climate data was important for predictive model performance [23,24,26]. This finding could indicate that the inclusion of climate data should be considered for disease outcomes related to outbreak status or spread of disease. Additionally, climate data may only be important for diseases in which environmental spread is of particular importance.

Laboratory testing data were included in several articles as inputs for predictive models [23,24,25,26,27,32,33]. These type of data are not as easy to obtain as the demographic and climate data. Climate data are easily obtained from many sources automatically and at a low cost through automatic programming interface (API) calls. Demographic data are often recorded on many levels of an operation for book-keeping purposes or to evaluate morbidity and mortality over time. Laboratory testing data are often not contemporaneous with other data. Time must be allowed for running a laboratory test and reporting the outcome. This can introduce errors. Additionally, the application of a predictive model is often desired at the time an intervention can be instated; thus, waiting for a test result is not always practical. Despite these limitations, laboratory testing may provide useful information and increase the accuracy of a predictive model depending on the test and predictive model application.

Several articles reported using images as input data for disease outcome predictions [28,34,35,36,37]. Image classification with machine learning has become a powerful tool in both veterinary and human medicine [38,39]. Additionally, studies report using images for the identification of individual animals [40,41]. Images or video data can capture data in a form that cannot otherwise be conveyed in a table. For example, an image of a single animal’s face may capture nasal and ocular discharge as well as drooping ears, all of which could indicate illness. Furthermore, an image may be more easily obtained than other types of data such as weight. In this example, it is easier to take a picture of an individual animal on pasture than it is to weigh that animal on a scale. This unique data source is also easy to collect with the right tools and, as technology improves, obtaining high quality images becomes less cost-prohibitive. A limitation of this data source is the required storage space needed to utilize it in machine learning tasks. Additionally, models trained on image data may require longer processing times to provide predicted outcomes, thereby limiting their utilization to assist with management decisions in real time.

Although not investigated in the studies covered by this literature review, on-animal and on-farm sensors are becoming an increasingly more common form of technology used to aid in animal health production monitoring. These sensors collect data automatically about individual animals, such as core temperature, feed and water intake, and ambulation. These sensors operate differently than the predictive models constructed in the articles selected for this review in that they measure a baseline for an individual animal and notify the user of any remarkable deviations from this baseline. This learning approach is called “unsupervised.” The predictive models mentioned in the current review utilize a “supervised” learning approach in which the algorithms are given labeled data where labels are pre-determined by the user [42]. Both unsupervised and supervised learning techniques can provide powerful and accurate models.

## 6. Machine Learning Algorithms

A wide variety of machine learning algorithms were developed to predict cattle health outcomes in the selected studies. They were categorized into four groups: Bayes-based, linear, neural network, and tree-based models. Brief summaries of these algorithms are provided below.

### 6.1. Bayes-Based Models

Bayesian predictive models use Bayesian inference to predict future outcomes. Bayes’ theorem is a model for combining prior experience with current evidence to draw a conclusion about the probability of an outcome or event [43]. The application of Bayes’ theorem to machine learning involves utilizing the observed data (usually in the form of a training dataset) to generate a prior distribution; then conditional likelihoods are generated for each outcome class. A posterior probability is then computed for each outcome class, and these are then used to predict the class with the highest posterior probability. A benefit of Bayesian predictive models is that the user can obtain a probability distribution over the possible outcome classes if desired. Additionally, these models tend to reduce overfitting [44].

### 6.2. Linear Models

This broad category includes all algorithms with a linear model background. This includes models like logistic and linear regression as well as linear discriminant analysis. Linear techniques are widely used and well-understood statistical models. These algorithms can be used to model both categorical and continuous outcomes. While they may appear simple, linear models can be applied to complex, multivariate analysis and are capable of handling complex data in the context of predictive modeling [45].

### 6.3. Neural Networks

Neural networks are named after the human brain due to their ability to recognize complex patterns using hidden layers and weights. Their approach to prediction is often called a “black box” due to the complex nature of the parameters provided as an output [46]. Their usage is particularly popular in fields with complex data (such as human and veterinary medicine), artificial intelligence software, and image data [47].

### 6.4. Tree-Based Models

Tree-based models included both decision trees and random forests. Both of these algorithm types are popular for classification prediction [48]. Decision trees are named for their decision-making structure, in which a “root node” represents the entire population of interest and “decision nodes” branch from the root node to represent the input variables splitting at specific criteria. Branches connect these decision nodes to “leaf nodes”, which represent the final outcome [49]. Random forests consist of multiple decision trees that are constructed from random subsets of the entire dataset. The outcomes of the many decision trees are then used by the algorithm to vote for a final prediction [50]. Tree-based models are unique in their ability to manage noisy data that may not follow a linear correlation pattern. Additionally, ensemble methods such as boosting and bagging are popular additions to these models to improve performance [48].

## 7. Imbalanced Data

The prevalence of an outcome, especially a disease-related outcome, should always be considered in the context of predictive model evaluation. Oftentimes, the outcome of interest may be a rare one, with low prevalence; however, the rare outcome is of interest to model due to significant economic or population health implications. The result is predictive models trained on imbalanced datasets. Evaluation of model performance on a single metric alone (such as accuracy) is not appropriate in such cases as this does not provide the full breadth of the model’s performance [13]. For example, a model trained on a dataset in which the prevalence of an outcome of interest is 5% may favor prediction of the negative outcome because it results in a high accuracy (95%). If that model is evaluated on accuracy alone, it appears to be a great model. However, upon evaluation of sensitivity (0%), it becomes clear that the model is predicting every row to be negative, which is not useful if the outcome of interest is presence or absence of disease.

This imbalance of the outcome of interest is apparent in the current review. Prevalences for disease outcomes in this study ranged from 6.1 to 86.0%. The application of predictive models to disease outcomes for which the positive class is rare may result in a tendency of the predictive model to favor selection of the majority class to improve accuracy [51]. In these cases, the predictive model may boast high accuracy but fail to detect any of the truly positive cases, resulting in a very low sensitivity. In applications in which disease detection is important, such a predictive model becomes useless. Several articles report high prevalences, which may have resulted in the opposite phenomenon, high sensitivity values while sacrificing specificity [23,24,25,26,37].

An additional consequence of utilizing imbalanced data is captured by the trends seen in Figure 2 and Figure 3. Both the NPV and PPV are metrics influenced by the sensitivity and specificity of the model as well as the prevalence of the outcome of interest. Oftentimes, the prevalence of disease-positive individuals tends to be lower than that of disease-negative individuals [9]. Very high NPVs and low PPVs demonstrate that models predicting a negative outcome have a higher probability of being correct and models predicting a positive outcome have a lower probability of being correct. Instances in which the prevalence of disease-positive individuals is higher would result in decreasing NPVs and increasing PPVs regardless of sensitivity and specificity—which are metrics that remain the same regardless of the prevalence of the outcome of interest.

Class-imbalanced data are perhaps the biggest challenge in the application of predictive models to determine health outcomes in beef cattle. There exist several strategies to cope with this challenge. Several machine learning approaches have been developed specifically to address class imbalance, such as ensemble methods like random forest or boosting [52,53]. Additionally, balancing of the outcome classes prior to testing using over- or undersampling techniques has also been described. Two articles included in the current review reported on the differences in model performance when utilizing a balancing technique in comparison to the baseline prevalence. The results demonstrated no improvement in predictive model performance metrics [16,19]. Other methods such as hybrid over- and undersampling, cluster-based, and distance-based methods also exist and have demonstrated promise in other fields [13]. Efforts should be made to address class imbalance either through the application of an ensemble method or a balancing technique in the application of predictive modeling to health outcome determination.

## 8. Conclusions

This review sought to provide a summary of the current literature evaluating the performance of machine learning techniques to predict health outcomes and disease occurrences in the beef production sector. There has been increased interest in the application of machine learning to improve health and productivity in cattle. The dairy sector of the industry remains ahead of the beef sector in applying predictive model technology. As the beef sector, in addition to the remainder of the livestock production industry, moves forward with machine learning integration, there are several important considerations, as revealed by the current review. First, selection of input data should balance utility in the model, ease of collection, and quality. Contribution to predictive model performance should be measured if possible and evaluated in the context of collection costs. Efforts should be made to remove data which could provide misleading information. Second, evaluation of predictive model performance should include several variables and consider the prevalence of the outcome of interest in order to avoid the development of predictive models that favor a single outcome class. Finally, the challenges of class-imbalanced data should be addressed with algorithm selection and/or the use of a balancing technique during training.

## Figures and Tables

**Table 1:** Inclusion and exclusion criteria applied to articles for literature review selection.

Inclusion	Exclusion
Primary research article	Narrative or systematic literature review, meta-analysis, conference abstracts, theses, books, and book chapters
Data used from dairy or beef cattle	Data used from small ruminants, poultry, companion animals, rodents, reptiles, equine, or other atypical bovids (bison, water buffalo, yaks, etc.)
Data must be at the individual animal or group/herd level	Simulated data
Outcomes of interest are related to a specific disease or condition or overall morbidity or mortality Pregnancy loss considered a disease	Outcomes of interest are related to production performance such as carcass characteristics, milk yield, pregnancy, weight gain, etc.
Machine learning/predictive modeling is utilized to anticipate the outcome of interest	Inferential statistics are used to determine an association between the outcome and predictor variables

**Table 2:** Description of data collected from articles included in narrative literature review.

Variable Name	Description
Title	Title of the research article as published
Authors	Last name and initials of all authors
Year	Year of publication
Journal	Title of journal in which article was published
Production_System	Broad category of animal type/use referred to in the article: beef, dairy, or both
Study_Arm	Letter to indicate study arm of the article
Outcome_Category	Word or phrase to describe the broad disease category being investigated in the article, for example, lameness or respiratory disease
Image_yn	Describes if images or video data were used to train the models
Climate_Data_yn	Describes if weather data were used to train the models. Includes data such as temperature, humidity, wind speed, etc.
TestResults_yn	Describes if laboratory test results were used to train the models. Includes data such as complete blood count results, intradermal tuberculosis testing, etc.
Demographic_Data_yn	Describes if demographic characteristics were used to train the models. Is further split into three different hierarchies: operation, group, and individual. Includes data such as number of animals on an operation, average weight of a group, rectal temperature of an individual, etc.
Number_EU	Number of experimental units in the training dataset given to the model
EU_Type	Type of experimental unit used to describe data hierarchy, for example, farm or individual animal
Algorithm	Machine learning or predictive model used in the study arm, as a broad category, for example, tree-based, linear, etc.
Accuracy, %	Measure of number of correctly classified units over total classified units
Sensitivity, %	Proportion of the truly positive individuals (diseased) that were identified as positive by the model
Specificity, %	Proportion of the truly negative individuals (disease-free) that were identified as negative by the model
Positive predictive value (PPV)	Proportion of the model-identified positive individuals that were truly positive
Negative predictive value (NPV)	Proportion of the model-identified negative individuals that were truly negative

**Table 3:** Distribution of articles and study arms from literature review by input data type and disease outcome category.

Input Data Type	Disease Outcome Category	Count of Articles	Count of Study Arms
Image or Video	Other	3	14
	Respiratory Disease	1	16
	Foreign Animal Disease	1	5
Climate Data	Other	1	11
	Respiratory Disease	1	5
	Bovine Tuberculosis	4	32
Laboratory Testing Data	Other	1	30
	Respiratory Disease	1	1
	Bovine Tuberculosis	5	35
Operational Demographic Data	Respiratory Disease	4	51
	Foreign Animal Disease	1	3
	Bovine Tuberculosis	5	35
Group-level Demographic Data	Other	1	5
	Respiratory Disease	5	55
Individual Animal-level Demographic Data	Other	1	11
	Respiratory Disease	4	50

**Table 4:** Description of health outcomes and prevalence of health outcomes investigated by articles included in the literature review.

Disease Category	Health Outcome	Count of Articles	Count of Study Arms	Average Prevalence, % (Range ^a^)	Number of Training Rows ^b^
Respiratory Disease	Acute interstitial pneumonia	1	4	11.8 (10.7–12.9)	30.5
	Broncho-interstitial pneumonia	1	4	38.5 (35.4–40.9)	85.8
	Bovine respiratory disease	7	62	20.1 (6.1–51.9)	31,843.5
	Other pneumonia	1	4	13.7 (11.4–16.4)	37
Foreign Animal Disease	Foot-and-mouth disease	1	3	27.0	583
	Lumpy skin disease	1	5	38.6 (31.6–49.1)	1284.6
Bovine Tuberculosis	Bovine tuberculosis	5	35	13.6 (3.58–86.0)	46,204.3
Other	Heat stress	1	11	11.8	301
	Infectious bovine keratoconjunctivitis	1	1	19.5	4500
	Liver abscess	1	30	44.8	166
	Total morbidity	1	5	18.5	389
	Anaplasmosis	1	1	79.1	8100
	Babesia	1	1	85	2871

^a^ Ranges only available for health outcomes with more than one entry for prevalence. ^b^ For diseases with multiple study arms, number of training rows is averaged over study arms.

## Data Availability

No new data were created.
